# Differential Odour Coding of Isotopomers in the Honeybee Brain

**DOI:** 10.1038/srep21893

**Published:** 2016-02-22

**Authors:** Marco Paoli, Andrea Anesi, Renzo Antolini, Graziano Guella, Giorgio Vallortigara, Albrecht Haase

**Affiliations:** 1 University of Trento, Center for Mind/Brain Sciences, Rovereto, 38068, Italy; 2 University of Trento, Department of Physics, Trento, 38123, Italy

## Abstract

The shape recognition model of olfaction maintains that odorant reception probes physicochemical properties such as size, shape, electric charge, and hydrophobicity of the ligand. Recently, insects were shown to distinguish common from deuterated isotopomers of the same odorant, suggesting the involvement of other molecular properties to odorant reception. Via two-photon functional microscopy we investigated how common and deuterated isoforms of natural odorants are coded within the honeybee brain. Our results provide evidence that (i) different isotopomers generate different neuronal activation maps, (ii) isotopomer sensitivity is a general mechanism common to multiple odorant receptors, and (iii) isotopomer specificity is highly consistent across individuals. This indicates that honeybee’s olfactory system discriminates between isotopomers of the same odorant, suggesting that other features, such as molecular vibrations, may contribute to odour signal transduction.

The shape recognition theory of odorant reception^1^ relies on experimental evidence linking structural modifications chemically introduced in a known odorant, to changes in odour character^2^,^3^. Nonetheless, it fails to explain convincingly how molecules of completely different structure can be associated to the same odour character^4^, nor does it explain why structurally minor single-atom replacements can strongly alter odour character^5^,^6^. A model of odorant-receptor interaction relying exclusively on stereochemical properties of the ligand can be tested by measuring the reception of common odorants versus their isotope-labelled analogues, in which some or all hydrogen atoms have been substituted with deuterium. If individual receptors were to respond differently to different isotopomers of the same odorant, this would point to a weakness of the shape theory of olfaction. On the contrary, an identical representation would strongly support the theory. Isotopomers have identical molecular size, shape, and surface charge, slightly different diffusion and evaporation rates, and notably different molecular vibration frequencies. Thus, isotopomer pairs represent the most appropriate experimental paradigm to test the validity of the “lock-and-key” model of odorant reception, and have been a useful tool for both supporters^7^,^8^ and detractors^9^,^10^,^11^,^12^,^13^ of this theory. Recent behavioural experiments have revealed that fruit flies and honeybees can distinguish isotopomers of the same molecule^9^,^11^,^12^. Other studies have shown that humans are able to discriminate between isotopomers of the same odorant^10^,^13^. Yet, experimental evidence against isotopomer discrimination^7^,^8^ keeps the debate open. Behaviour, however, is a complex readout. Hence, we tested the perceptual distinguishability of common and deuterated odorants by analysing their neural representation in the primary coding and processing centres of the olfactory circuit: the antennal lobes (ALs)^14^. Within the ALs, each odorant is represented by a conserved spatio-temporal pattern of glomerular activation^15^. Here, we investigated and compared the response patterns elicited by four common odorants: 1-octanol (OCT-h), benzaldehyde (BZA-h), acetophenone (ACP-h), and isoamyl acetate (ISO-h), and their deuterated isotopomers (OCT-d_17_, BZA-d_5_, ACP-d_8_, ISO-d_3_), revealing that stereochemically identical molecules are differently coded within the honeybee olfactory circuit.

## Results

**Figure 1:**
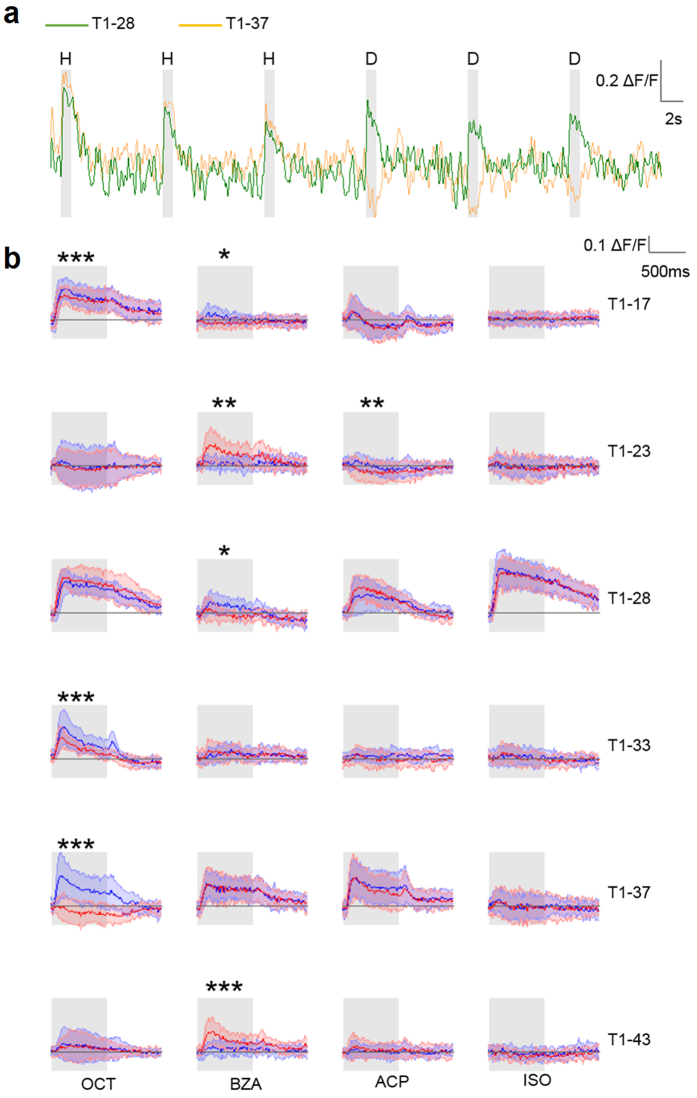
Induced glomerular responses. (**a**) Activity profiles of glomerulus T1-37 and T1-28 measured in one individual during 3 presentations of OCT-h (H) followed by 3 presentations of OCT-d_17_ (D). (**b**) Mean odorant response profiles ± s.d. of 6 exemplificative glomeruli (rows) to four odorants (columns). Response profiles to the common isoforms are plot in blue, to the deuterated isoforms in red; grey shadows indicate stimulus delivery. Significant differences are indicated (paired-sample *t*-test, *p < 0.05, **p < 0.01, ***p < 0.005; *N* = 11 for BZA and ISO, *N* = 12 for ACP and OCT). Odorant abbreviations are 1-octanol: OCT; benzaldehyde: BZA; acetophenone: ACP; isoamyl acetate: ISO.

**Figure 2:**
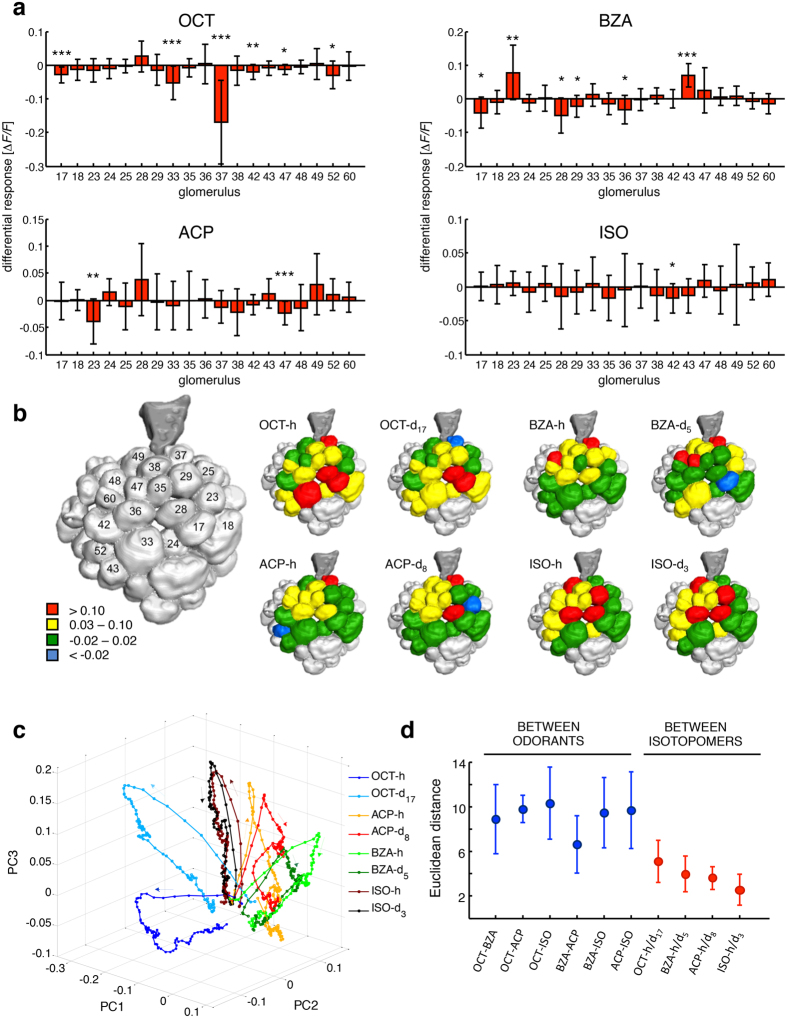
Odorant response maps. (**a**) For each subject, the difference between the responses to H- and D-isotopomer was calculated and mean differences across subjects ± s.d. are plotted. Significant differences are highlighted (paired-sample *t*-test, *p < 0.05, **p < 0.01, ***p < 0.005; *N* = 11 for BZA and ISO, *N* = 12 for ACP and OCT). (**b**) Graphical representation of odorant-induced response maps. Glomerular responses were classified in 4 groups: red, high activation: −Δ*F*/*F* > 0.10; yellow, low activation: 0.03 ≤ −Δ*F*/*F* ≤ 0.10; green, no activity: −0.02 ≤ −Δ*F*/*F* ≤ 0.02; blue, inhibition: −Δ*F*/*F* < −0.02; glomeruli in grey were not considered in the analysis. (**c**) PCA of AL response dynamics elicited by the odorants during 1 s stimulus exposure and 1 s post-stimulus phase. Arrows indicate the temporal order of signal build-up and decay. (**d**) Mean Euclidean distances ± s.d. between different pairs of odorants (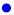) and within pairs of isotopomers (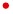). Odorant abbreviations are 1-octanol, OCT; benzaldehyde, BZA; acetophenone, ACP; isoamyl acetate, ISO.

Odour response patterns were measured in a subset of 19 glomeruli by monitoring intracellular calcium concentration in the presence of controlled olfactory stimulation^16^. Two representative single-glomerular responses are shown in Fig. 1a. An identical response was elicited by both OCT isotopomers in glomerulus T1-28, whereas isotopomer-specific activation was encountered in glomerulus T1-37 in the same individual during the same experimental session. This analysis was extended to a group of 12 honeybees and the mean glomerular response profiles were calculated (Fig. 1b). Remarkably, the differential response of glomerulus T1-37 to OCT-h versus OCT-d_17_ observed for one individual was conserved across the analysed group. Next, odorant-elicited responses were determined for the whole glomerular subset, and differential response maps were characterized across all individuals (Fig. 2). For OCT, several glomeruli were responsive to both isotopomers, but with significantly different intensity (*e.g.* T1-17, T1-33). Most striking was the isotopomer-specificity of glomerulus T1-37 (Figs 1 and 2), which showed opposite responses for the two isotopomers. For BZA isotopomers, significantly different responses were induced in six of the analysed glomeruli (*e.g.* T1-17, T1-23, and T1 43). For ACP, an isotopomer-specific response was observed in glomeruli T1-47 and T1-23, the latter significantly inhibited upon exposure to ACP-d_8_. Finally, for ISO, only T1-42 showed a significant preferential response to the heavy isotopomer.

To investigate the dynamics of the olfactory responses, we considered for all compounds the change of the elicited response maps averaged across animals during a time span of 2 s, comprising 1 s of stimulus delivery plus 1 s post-stimulus. To reduce the dimensionality of the multi-glomerular space, we performed a principal component analysis. The first three principal components appeared to describe 93% of all the data variances, and were used to visualize the response dynamics (Fig. 2c). Responses elicited by both ISO isotopomers were virtually coextensive, as expected from the small difference between relative odorant-response maps (Fig. 2a,b). In contrast, trajectories of the responses to OCT-h/d_17_ were clearly separated in the olfactory coding space. Odorant-response trajectories of BZA and ACP pairs showed intermediate results. Next, we computed the Euclidean distances between tested odorants and within isotopic pairs (Fig. 2d). Odorants with very different molecular structures elicited responses with Euclidean distances between 9 and 10. Benzaldehyde and ACP-h, which share a benzene ring, showed a smaller distance of about 6. Euclidean distances between isotopomer pairs of the same odorants ranged from 3 to 5, with the distance between OCT-h and OCT-d_17_ comparable to that between BZA-h and ACP-h. This quantitative analysis further supports the hypothesis that common and deuterated isotopomers are distinguished by olfactory receptors.

**Figure 3:**
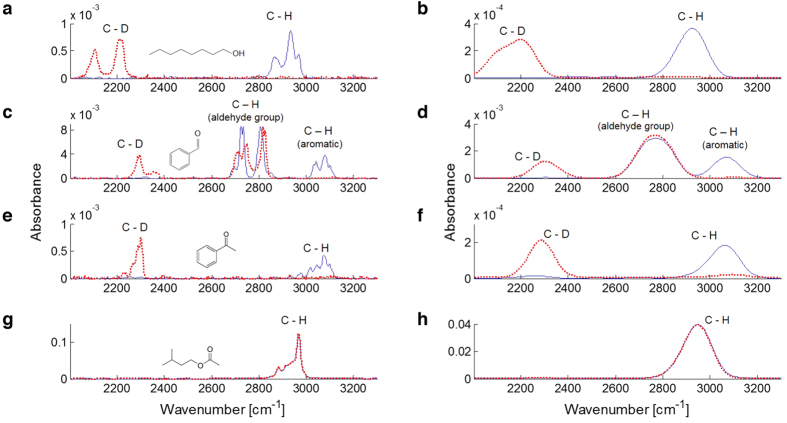
Vibrational spectra comparison. OCT (**a**,**b**), BZA (**c**,**d**), ACP (**e**,**f**), and ISO (**g**,**h**) spectra within the 2000–3300 cm^−1^ region unfiltered (left column), and broadened (right column). Spectra show absolute absorbance after air baseline subtraction. The different orders of magnitude are due to the different volatility of the compounds. Common (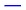) and deuterated (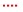) isotopomers’ profiles are presented. Skeletal formulas of common odorants are shown. Odorant abbreviations are 1-octanol: OCT; benzaldehyde: BZA; acetophenone: ACP; isoamyl acetate: ISO.

Although structurally identical, isotopomers differ slightly in molecular weight, have a slightly different susceptibility to enzyme digestion, and a dissimilar molecular vibration profile. Because of the open debate on a possible role of intramolecular vibrations in odour signal transduction, the so-called vibrational theory of olfaction^5^, we measured the vibrational spectra of the tested odorants in gaseous form, thus mimicking real stimulus delivery conditions. The molecular vibration spectra were analysed via FTIR spectroscopy in a range from 500 to 3300 cm^−1^. This spectral range can be subdivided into the fingerprint region, from 500 to 1500 cm^−1^, which contains a variety of bending modes at high peak-density, and a region of higher wavenumbers (>1500 cm^−1^), containing only a few well-separated vibrations due to bond stretching. A potential biological spectrometric mechanism operating at the bee’s body temperature of up to 37 °C is limited by thermal fluctuations to resolutions of the order of *kT*/*hc* ≈ 215 cm^−1^. Adding the effect of thermal fluctuations flattened the spectral differences throughout the fingerprint region (Supplementary Figure S1 online), suggesting that thermal noise could hide many of the subtle differences from a low-resolution biological spectrometer. In contrast, the visibility of the well-separated absorbance peaks of the upper part of the spectrum was not compromised, allowing these vibrational modes to be deciphered even by a resolution-limited mechanism (Fig. 3). Since the most pronounced effects of the deuteration is the shift of the C-H modes from 2800–3000 cm^−1^ to the 2000–2200 cm^−1^ of the C-D modes, we focused on the region between 2000 and 3300 cm^−1^. Notably, we observed that the IR frequencies of highly deuterated molecules (OCT, BZA, ACP) were clearly separated from their hydrogenated analogues, whereas the IR spectra of the isotopomers of the weakly deuterated ISO-d_3_ were almost completely overlapping, since the relative contribution of the C-D stretch vibration to the IR absorption was negligible.

## Discussion

Hereby, we report that isotopic isomers are differently represented in the honeybee antennal lobe, supporting behavioural experiments showing that fruit flies and honeybees can discriminate isotopomers of the same odorant^9^,^11^. Also, we wish to underline that the majority of the glomeruli responded in a similar fashion to the presentation of both isotopomers of an odorant, suggesting little influence of the deuteration on the dynamic guiding the arrival of the odorants to the olfactory receptors (ORs). However, some glomeruli displayed a robust isotopomer-specificity, which was consistently observed among the tested honeybees (Figs 1 and 2a,b). Furthermore, diverse isotopomer-sensitive glomeruli were identified for each tested odorant. This suggests that such shape-independent discrimination ability is not a peculiarity of certain receptors, but rather a general feature of the ligand-receptor interaction mechanisms, that may complement the shape-recognition-based odour coding. Glomerulus T1-23 shows an odorant-induced response, which is rather interesting, for it shows differential response for both BZA and ACP isotopomers, but with opposite preferences (Fig. 1b). Indeed, this glomerulus displays higher activation upon exposure to the deuterated than to the common BZA, whereas it shows a stronger response to common ACP than to its deuterated isotopomer. This is interesting if considering that BZA and ACP share a very similar structure (see Supplementary Figure S2 online), and are therefore expected to elicit a similar response profile. Indeed, the hydrogenated form of both odorants evokes a negligible response in glomerulus T1-23. Instead, while BZA-d_5_ triggers a positive response, the same glomerulus is significantly inhibited by exposure to ACP-d_8_. Considering that the only structural difference between the two compounds is in the acetyl carbon present in the ACP, and that such difference alone does not trigger a differential response in glomerulus T1-23, it is licit to speculate that the different responses reported in this analysis could be due to the isotope sensitivity of the corresponding OR.

**Figure 4:**
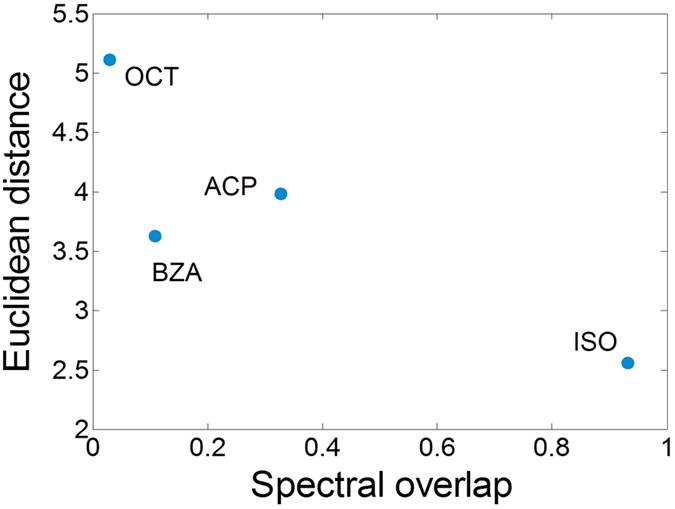
Glomerular activation versus vibrational spectra similarity. Euclidean distances of the average odorant response maps between pairs of isotopomers are plotted against their differences in spectral overlap in the spectral window of 2000–3300 cm^−1^. Odorant abbreviations are 1-octanol: OCT; benzaldehyde: BZA; acetophenone: ACP; isoamyl acetate: ISO.

Next, we reproduced the olfactory stimulation conditions and measured the vibrational spectra of common and deuterated isotopomers, showing that the spectral region >2000 cm^−1^ presents a limited number of vibrational resonances, which are well resolved even after thermal broadening. Recently, it has been suggested that this high-wavenumber window provides good correlations between vibrational frequencies and odour characters, both in experimental and computational approaches^9^,^12^,^13^,^17^,^18^, even though potentially informative peaks were also found at lower frequencies^10^. To evaluate the hypothesis that vibrational modes may be informative for odorant-dependent receptor activation, we plotted, for each pair of isotopomers, the Euclidean distances between their glomerular response maps as a function of their vibrational spectra overlap (Fig. 4). This analysis revealed that, at least for the set of odorants here considered, similarity in vibrational spectra appear to mirror perceptual similarity. Common and deuterated isoforms of OCT, BZA, and ACP, whose spectra show clear non-overlapping features, were represented by significantly different response patterns in the honeybee AL. Conversely, common and deuterated ISO, with almost indistinguishable spectra, were represented as the same odour. Notably, this overlap is the simplest possible quantification of spectral differences, and does not allow any claim on the relationship between vibrational spectra and receptor responses.

Recently, Block *et al.*^8^ challenged the vibrational theory of reception in a clean *in vitro* system, and showed that common and deuterated isotopomers of the tested odorants have identical affinities to all tested ORs, thus suggesting that H/D substitution holds no significant effect on odorant-receptor interaction in a biological system. Such conclusions were obtained by expressing human and murine ORs in Hana3A cells, a heterologous cell system lacking many accessory cofactors, and exposing them to several common and deuterated odorants, among which benzaldehyde and acetophenone. Besides common issues related to testing biological principles on *in vitro* models, it must be noted that the protein receptor identified in insects ORNs are only distantly related to mammalian G-protein coupled receptors, and their dependency on G-proteins is still debated^19^,^20^. For these reasons, it is doubtful whether such findings can be considered a conclusive evidence against differential reception of isotopomers. However, they clearly show that different degrees of deuteration do not affect the odorant-receptor binding process. This may suggest that any isotopomer specific effect on odour-dependent activity perception may not be due to a different biochemical interaction with the receptor, but to different biophysical properties of the odorants, or to isotopomer-selective perireceptor events. Also, we assume that the contribution of molecular vibrations, or of any other biophysical property, is likely secondary to shape recognition. Indeed, in our observations, the majority of the investigated glomeruli do not show any isotopomer specificity. Still we reported significant isotope sensitivity in 15 of 76 tested glomerular responses, albeit while using only a small subset of the full odorant repertoire.

Furthermore, the different molecular masses of hydrogen and deuterium atoms directly affect the C-H/D bond energy. Hence, degradation reactions involving C-H/D bond breakage may occur at different rates. If so, this could result in a different sensitivity of the odorant to odorant-degrading enzymes (ODEs). This is a crucial issue that needs to be investigated, not only to probe the mechanism that allows isotopomer discrimination, but also to clarify the molecular events leading to odour perception. In this regard, the major class of enzymes linked to odorant degradation in insects is cytochromes P450^21^. Guengerich *et al.* analysed in detail the kinetic isotope effect (KIE) of several P450 enzymes, reporting a *KIE *≥ 1 (*KIE* = *k*_H_/*k*_D_, where *k*_H_ and *k*_D_ represent the rate of C-H and C-D bond breakage)^22^. This indicates that cytochromes P450 do not degrade deuterated compounds faster than common ones. Therefore, the decrease in glomerular response observed upon exposure to the deuterated versus the common isotopomer should not be due to a faster degradation of the heavy isoform (Figs 1 and 2a). Indeed, the opposite isotope dependency detected in glomerulus T1-23 for two different odorants, cannot be explained by a preferential degradation of hydrogenated or deuterated compounds. Moreover, Chertemps *et al.* recently reported on the role of the EST-6 esterase in *Drosophila* perireceptor environment, suggesting that ODEs have an important influence on signal duration, but no significant effect on signal onset or maximum amplitude^23^.

A final concern regarding the experimental proof of isotopomer-sensitivity lies in the possibility that contaminations of the tested odorants may give rise to differential responses. In this study, chemicals of the highest-available purity were employed, and all vials’ headspace quality was determined by GC analysis to >99.2% purity, and the degree of deuteration was verified by ^1^H-NMR to be ≈99% (Supplementary Table S1 online). Nevertheless, high specificity of an OR for a certain contaminant could potentially alter the odorant response map. However, since single glomeruli were observed even to invert their activation pattern between common and deuterated isotopomers, the contribution of any potential trace of impurity (further diluted in mineral oil) would have to completely overshadow the nominal odorant. Such effect would require receptor sensitivity 3 to 4 orders of magnitude higher in favour of the impurity, being its concentration about 1000 times smaller. This seems highly unlikely considering that all differences reported here for multiple glomeruli would have to be caused by this specific scenario.

In summary, we provided the first neurophysiological evidence of differential neural coding of isotopomers. Also, in light of the recent literature on ODEs, and considering that odour transduction in insects occurs in ≈2 ms^24^, it seems unlikely that perireceptor events could be responsible for the reported isotope sensitivity. Finally, we performed direct measurements of the vibrational spectra of each odorant, and reported that spectral differences among isotopomers well reflect differences in the elicited neural representation. In conclusion, common and deuterated odorants elicit different physiological responses within the honeybee antennal lobe. Nevertheless, the mechanism behind isotopomer recognition remains elusive, and may rely on different biophysical properties such as molecular weight, enzyme susceptibility, vibrational spectra, or, possibly, on a combination of them.

## Methods

### Animal model

Forager honeybees (*Apis mellifera*) were collected between May and August from outdoor beehives. Bees were kept indoors and fed with 50% sucrose solution for a maximum of 24 h before staining procedure.

### Staining and preparation procedure

Animal preparation for calcium imaging consists of a two-day procedure^25^. Briefly, on the first day, bees collected from the beehive were placed on a custom-made Plexiglas mount, where thorax and abdomen were free to move but the head was stabilized with soft dental wax (Deiberit 502, Siladent). Both antennae were temporarily fixed facing forward with a small drop of Eicosane (Sigma-Aldrich) to avoid damage during preparation. A small window in the head cuticle was opened. Glands and tracheae covering the site of injection were displaced. The tip of a borosilicate glass needle covered with Fura-2 dextran (Life Technologies) was inserted between the mushroom body calices, below the alpha lobe, where medial and lateral projection neuron tracts cross, and kept in place until the dye crystal was dissolved. After the staining procedure, the head capsule was closed to prevent brain from drying. Stained honeybees were kept overnight at 20 °C in the dark to allow dye diffusion. On the second day, the cuticle was re-opened, glands and tracheae were removed, and the antennal lobes were exposed. Haemolymph in excess was removed, and the brain was covered in transparent Kwik-Sil (World Precision Instruments).

### Olfactory stimulation

Olfactory stimulation was performed with a custom-built, computer-controlled olfactometer inspired by an odorant delivery device used by Szyzska and colleagues^26^. The mechanical apparatus comprises 8 identical channels, each loaded with two Teflon-sealed glass vials: one odorant, one blank. Each odorant vial contained 1 mL of mineral oil-diluted odorant; each odourless vial contained 1 mL of mineral oil only (Sigma-Aldrich). All channels received the same airflow and converged into a nosepiece facing the honeybee. Solenoid valves (Lee Products Ltd) were placed downstream of the odorant vials to switch the airflow between odorant and blank channels. Continuous air suction behind the bee cleared residual odorant. The array of valves was operated via custom electronics, which were computer-controlled by a PCIe-6321 multifunction board (National Instruments). LabVIEW-based custom-made software synchronized stimulus delivery and microscopy acquisition with a precision of <10 ms. Four common odorants (Sigma-Aldrich) and their deuterated isotopomers (CDN Isotopes) were employed for the olfactory stimulation experiments. To vary the degree of similarity, different grades of deuteration were chosen: for ACP, a fully deuterated isotope; strong deuteration for BZA (the complete benzene ring) and OCT (the entire octane chain); weak deuteration for ISO (one terminal acetyl group). Compounds specifics are: 1-octanol (OCT-h), purity > 99%, and 1-octanol-d_17_ (OCT-d_17_), CD_3_(CD_2_)_7_OH, purity 98.5%, isotopic enrichment 98.8%; benzaldehyde (BZA-h), purity > 99%, and benzaldehyde-2,3,4,5,6-d_5_ (BZA-d_5_), C_6_D_5_CHO, 99.9%, isotopic enrichment 99.7%; acetophenone (ACP-h), purity > 99%, and acetophenone-d_8_ (ACP-d_8_), C_6_D_5_COCD_3_, purity 99.4%, isotopic enrichment 99.3%; isoamyl acetate (ISO-h), purity > 99%, and isoamyl acetate-d_3_ (ISO-d_3_), (CH_3_)_2_CHCH_2_CH_2_OCOCD_3_, purity 98.6%, isotopic enrichment 99.6% (see Supplementary Figure S2 online for structural formula). Each odorant was freshly prepared weekly. BZA-h/d_5_ and ISO-h/d_3_ were presented in a 1:500 dilution in mineral oil; OCT-h/d_17_ and ACP-h/d_8_ were presented in a 1:250 dilution. During the experimental procedure, the single-odorant channels were opened 3 times for 1 s, each time followed by a 9 s-pause, and no signal adaptation was observed upon successive exposure to the same stimulus. Each animal was exposed twice to the same odorant via different channels to avoid a channel-specific effect. Thus, the final glomerular response measurement for a single honeybee was based on a total of 6 exposures from two channels carrying the same odorant.

### Two-photon optical imaging

The optical setup consisted of a two-photon microscope (Ultima IV, Bruker) combined with an ultra-short pulsed laser (Mai Tai Deep See HP, Spectra-Physics-Newport). The laser was tuned to 800 nm for Fura-2 excitation. Galvanometric mirrors allowed for fast and variable scanning. All images were acquired using a water-immersion objective (20x, NA 1.0, Olympus). The fluorescence was collected in epi-configuration. It was separated by a dichroic mirror (Chroma Technology Corp. filtered by a 70 nm band-pass filter centred at 525nm and detected by photomultiplier tubes (Hamamatsu Photonics). Optimal signal-to-noise ratio was achieved with laser powers of ≈10 mW without any sign of photobleaching, even during repeated exposure. Functional data acquisition was performed scanning along a custom 1D scanline at a rate of 50 Hz, repeated in 3 to 5 different imaging plains (Supplementary Figure S3 online). To avoid any bias due to anatomical and functional asymmetries^27^,^28^, all imaging experiments were conducted on the right AL.

### GC-MS analysis of standard compounds

Few drops of each standard compound were transferred into 7 ml clean sealed tubes and let to equilibrate. 300 μl of headspace were withdrawn with a gas tight syringe and injected into MS and FID injectors. Each syringe was washed 9 times with n-pentane (3 times from 3 different batches), 3 times with n-hexane, dried under vacuum for at least 10 min, and flushed with N_2_. Blank samples, injecting 300 μL of N_2_, were run before each injection to ensure a complete syringe cleaning. Samples were injected in splitless mode into Thermo Scientific Trace GC-DSQ II mass spectrometer (Thermo Fisher Scientific Inc.) equipped with a Flame Ionization Detector (FID), and controlled by Xcalibur 2.0.7 software (Thermo Fisher Scientific Inc.). The gas chromatograph was fitted with two DB5 60 m × 0.25 mm × 0.25 μm columns (Agilent Technologies). The carrier gas was helium at a constant flow of 1.4 ml/min for column connected to MS and of 1.9 ml/min for the one connected to FID injectors. MS transfer line temperature was set to 250 °C, while FID was set to 300 °C. The oven temperature was started at 50 °C and held for 0.5 min, then increased to 300 °C at 25 °C/min, and the final temperature was held for 0.5 min. The mass spectrometer quadrupole temperature was set to 150 °C, and the source to 230 °C. Electron ionization (EI) spectra at 70 eV were recorded in the range of *m/z* 50 to 500 for scan runs; detector gain was set to 0.3 × 10^5^ V. Peaks were integrated with Xcalibur 2.0.7 software on chromatograms obtained by FID, while MS data were used for compound identification. Gas chromatography profiles are reported in Supplementary Figures S4-S11 online.

### Optical data analysis

Calcium imaging data were acquired from a subset of 19 glomeruli innervated by the T1 receptor neuron branch^29^ from 11 (BZA and ISO) or 12 (OCT and ACP) bees. The analysis of the fluorescence data was performed by custom algorithms in MATLAB (MathWorks). Data post-processing, including background subtraction, normalization, and filtering of fluorescence-response curves, was performed automatically after acquisition to avoid any analytical bias. The standard honeybee brain atlas^29^ was used to assign fluorescence intensity variation dynamics along the scanline to specific glomeruli of the AL. Only superficial glomeruli were considered to avoid potential identification errors; non identifiable glomeruli were discarded. Quantitative response maps were based on the average fluorescence change between 200 and 600 ms after stimulus onset for each glomerulus. Since every individual was exposed to both common and deuterated isoforms of an odorant, a paired-sample *t*-test was performed to detect significant differences between glomerular activities in the presence of different isotopomers. The differential response was calculated as the mean within-subjects difference of the H- and D-isotopomer-elicited responses. Principal component analysis (PCA) was used to reduce the glomerular space dimensionality; the first three components described 93% of all signal dynamics (see Supplementary Figure S12 online for 2D PCA plots). Next, the Euclidean distance was calculated in the original multiglomerular space as a scalar measure for the odorant distinguishability as follows:


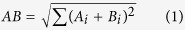


where *A*_*i*_ and *B*_*i*_ are the glomerular activities of glomerulus *i* in response to odorant *A* and *B* respectively, integrated over the 200–600 ms window from stimulus onset.

### IR spectroscopy

Molecular vibration spectra were measured via Fourier transform infrared spectroscopy (FTIR). The vial headspace was pumped into a 10 cm path-length gas cell (PIKE Technologies) within the FTIR spectrometer (Nicolet iS50) at constant airflow, as in the functional imaging experiments. To increase signal-to-noise ratio, the odorants were not diluted. For every compound, the absorbance was calculated from the background-subtracted spectra and area-normalized within every isotopomer pair. To quantify the differences between two spectra, the spectral overlap was calculated by integrating over the spectral differences, and normalized according to the following formula:


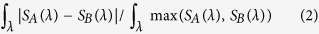


where *S*_*x*_(*λ*) is the absorbance of odorant *x* at wavelength *λ.* A similar approach was used to relate a spectral overlap to the similarity in molecules’ odour character as perceived by humans^30^. However, the algorithms for modelling electron-tunnelling spectra strongly emphasize the fingerprint region. In our measured IR spectra, the background absorption increases in this region, and deuteration-induced shifts are smaller. Together with a higher density of vibrational modes, the simple spectral overlap becomes non-informative at a low spectral resolution.

Thermal noise as present in the natural environment of olfactory receptors was simulated by convoluting the optical spectra with a Gaussian of *σ* = 100 cm^−1^ approximating thermal broadening at 37 °C, where *kT*/*hc* ≈ 215 cm^−1^ (Fig. 3 and Supplementary Figure S1 online).

## Additional Information

**How to cite this article**: Paoli, M. *et al.* Differential Odour Coding of Isotopomers in the Honeybee Brain. *Sci. Rep.* **6**, 21893; doi: 10.1038/srep21893 (2016).

## Supplementary Material

Supplementary Information
