# Supplementary material for: Differential Odour Coding of Isotopomers in the Honeybee Brain
Source: Sci Rep. 2016 Feb 22;6:21893. doi: 10.1038/srep21893 (PMC4762004; doi:10.1038/srep21893)
Supplement: Supplementary Information [file srep21893-s1.pdf]

Supplementary Information

Differential Odour Coding of Isotopomers in the Honeybee Brain

Marco Paoli[1,*], Andrea Anesi[2], Renzo Antolini[1,2], Graziano Guella[2], Giorgio Vallortigara[1], and Albrecht Haase[1,2,*]

[1]University of Trento, Center for Mind/Brain Sciences, Rovereto, 38068, Italy

[2]University of Trento, Department of Physics, Trento, 38123, Italy

*Correspondence and requests for materials should be addressed to M.P. (email: marco.paoli@unitn.it) and A.H. (email: albrecht.haase@unitn.it)

Supplementary Figure S1. Spectral overlap along the 500-2000cm^{-1} region with and without Gaussian filtering. OCT (**a**,**b**), BZA (**c**,**d**), ACP (**e**,**f**), and ISO (**g**,**h**) spectra without (left column), and with Gaussian broadening (right column). Y-axis values represent absolute values of absorbance after air baseline subtraction. The different orders of magnitude are due to the different volatility of the compounds. Common (—) and deuterated (—) isotopomers' profiles are presented. Skeletal formulas of common odorants are shown.

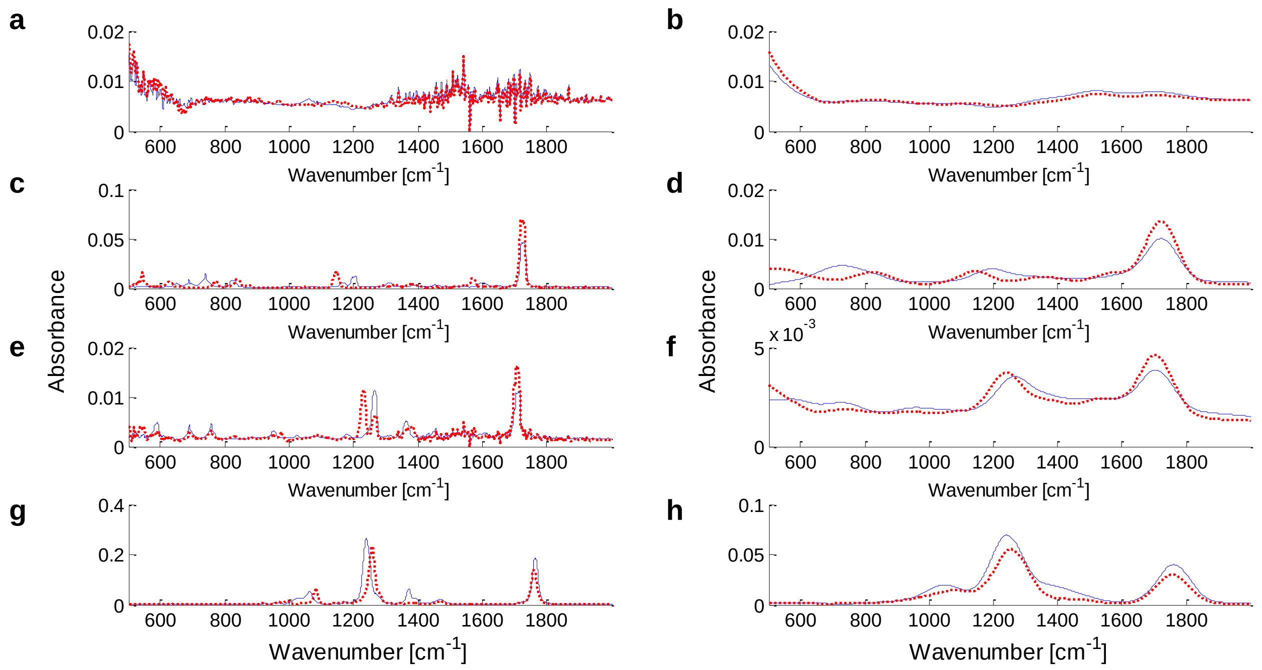

Supplementary Figure S2. Structural formulas of used odorants.

1-octanol

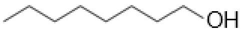

1-octanol-d_{17}

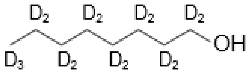

Benzaldehyde

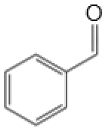

Benzaldehyde-d_5

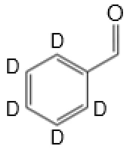

Acetophenone

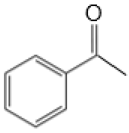

Acetophenone-d_8

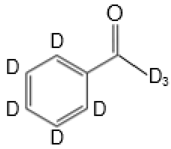

Isoamyl acetate

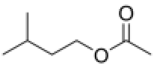

Isoamyl acetate-d_3

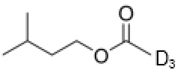

Supplementary Figure S3. Functional imaging data acquisition. (**a**) 3D reconstruction of a representative honeybee right antennal lobe. Glomeruli of T1 tract were identified according to the reference atlas. (**b**) Antennal lobe focal plane during acquisition at the two-photon microscope. Glomeruli are visible as round-shaped structures, and the white spiral-like trace indicates the selected scanning path of the laser (scanning time ≈20ms). (**c**) Fluorescence along the scanline (horizontal axis) was acquired in time (vertical axis). Synchronization with stimulus delivery allowed identification of odour-specific responses to common (H-) and deuterated (D-) isotopomers. For the purpose of example, activities of glomeruli T1-38 (red) and T1-28 (green) are highlighted.

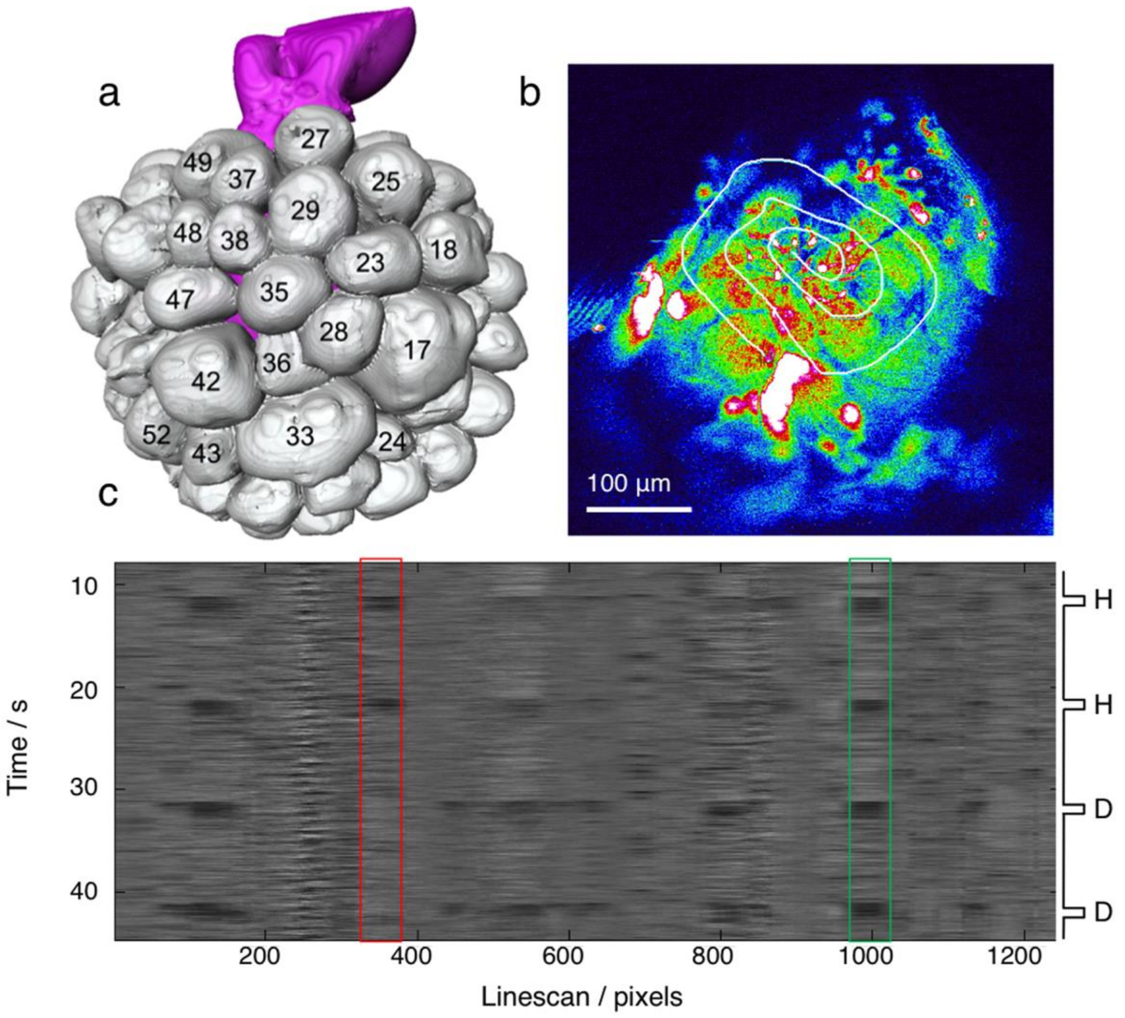

Supplementary Figure S4. Gas chromatography profile of acetophenone.

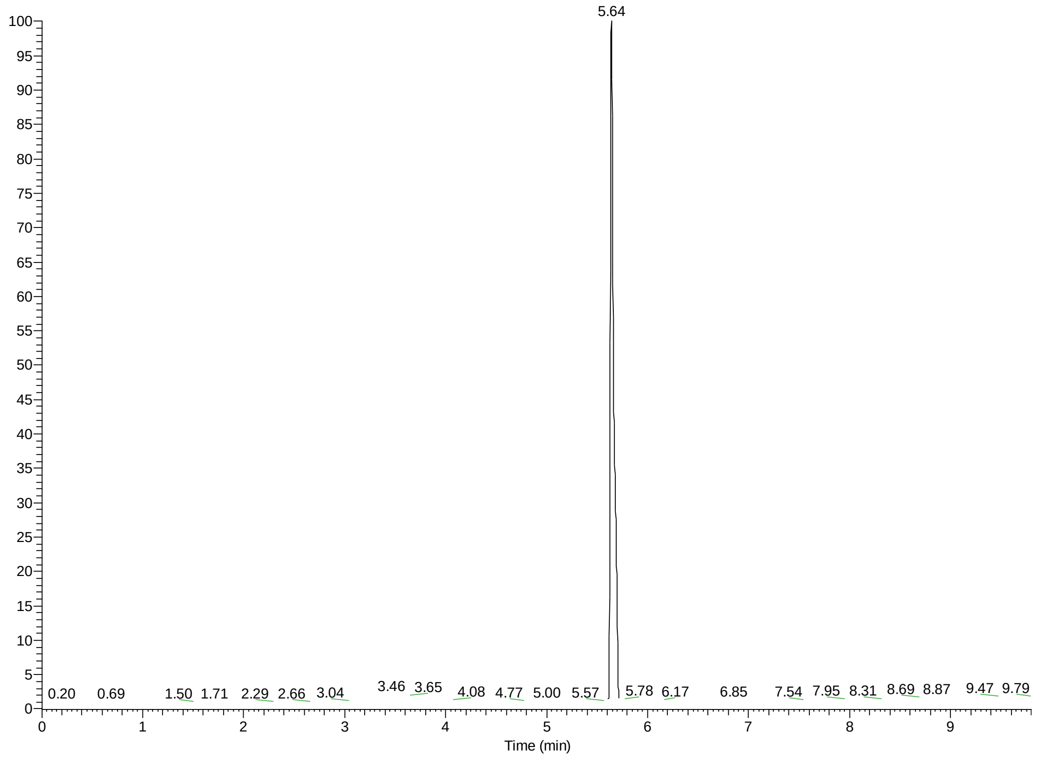

Supplementary Figure S5. Gas chromatography profile of acetophenone-d_8.

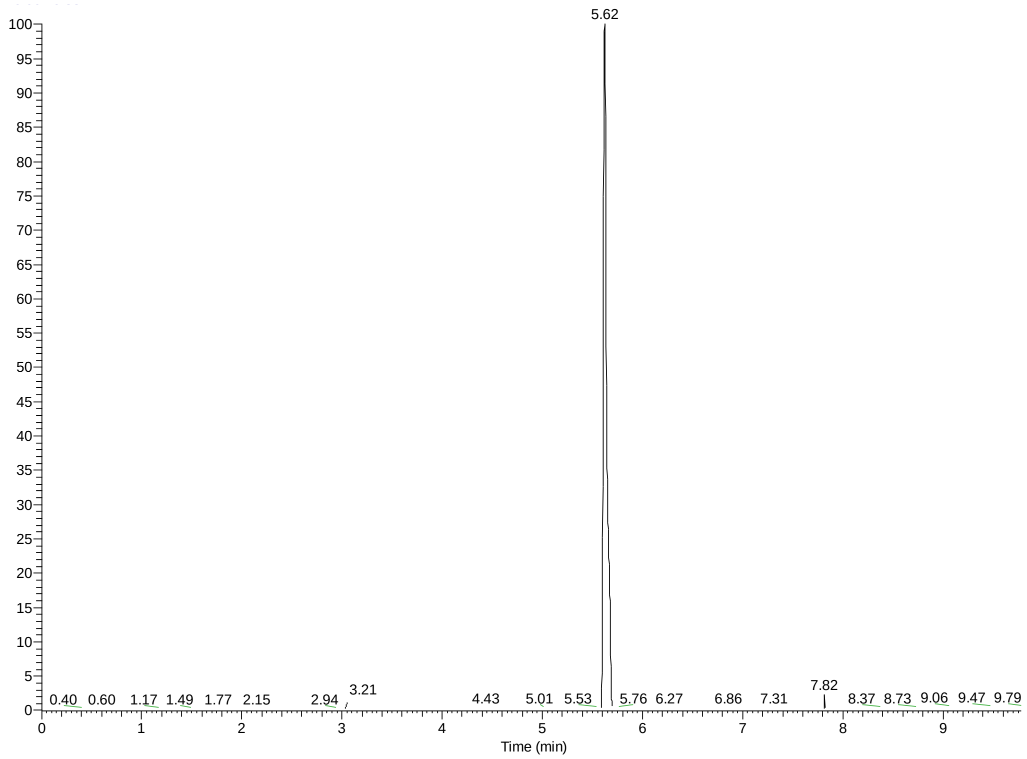

Supplementary Figure S6. Gas chromatography profile of benzaldehyde.

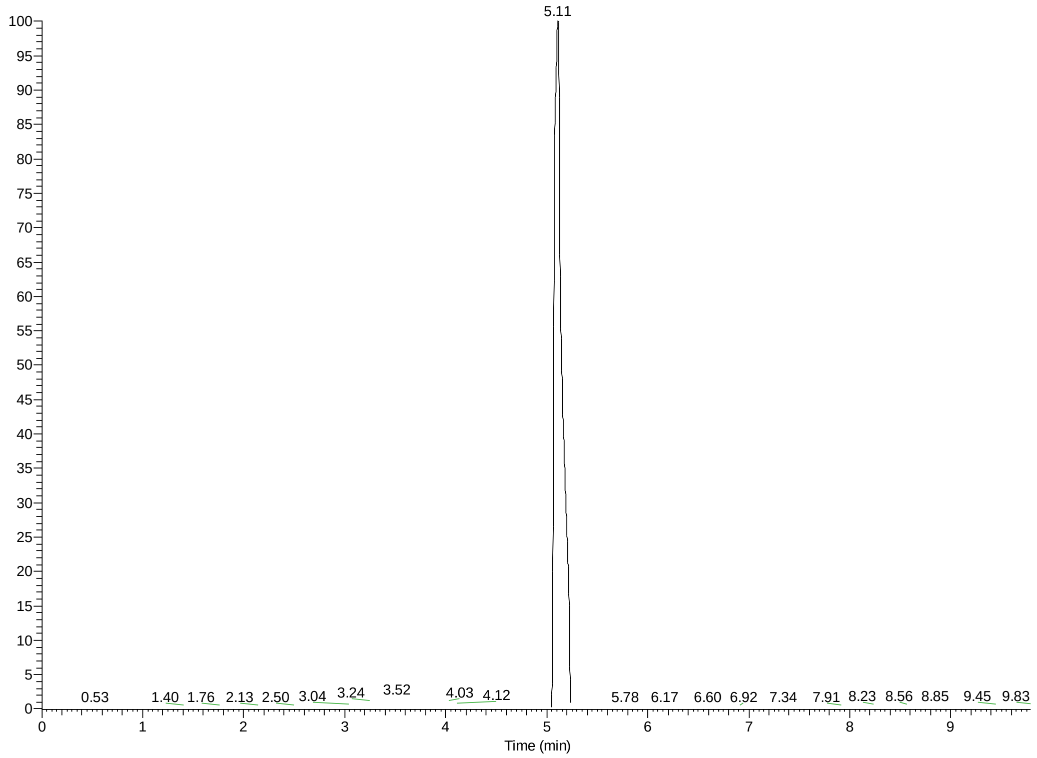

Supplementary Figure S7. Gas chromatography profile of benzaldehyde-d_5.

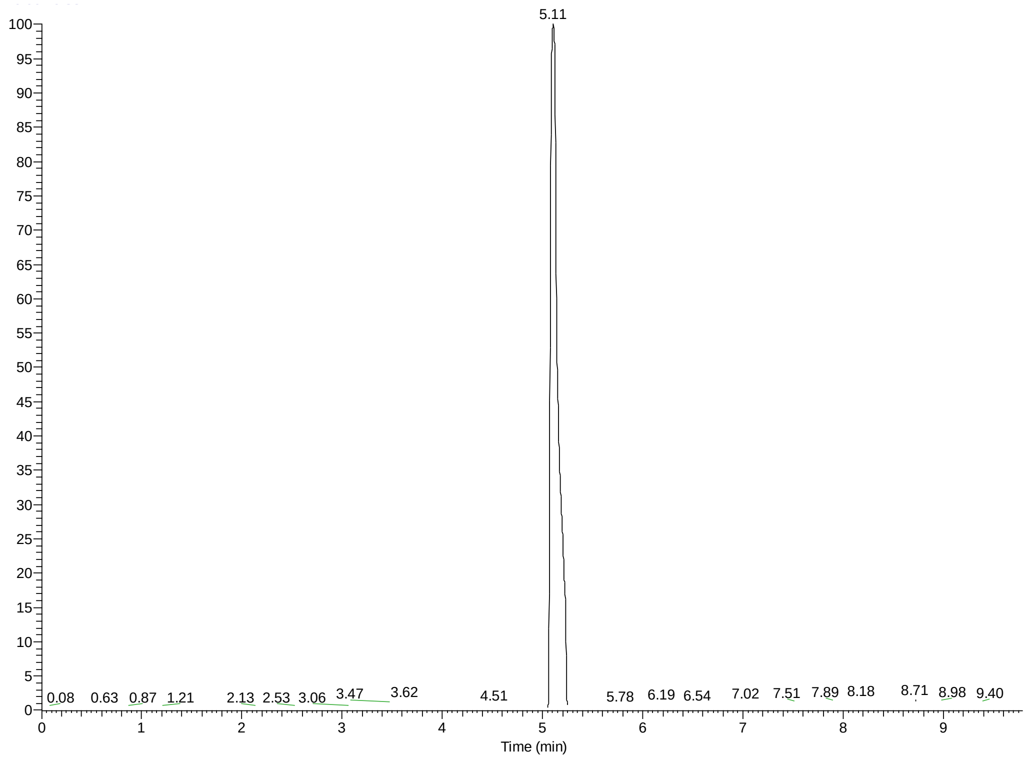

Supplementary Figure S8. Gas chromatography profile of 1-octanol.

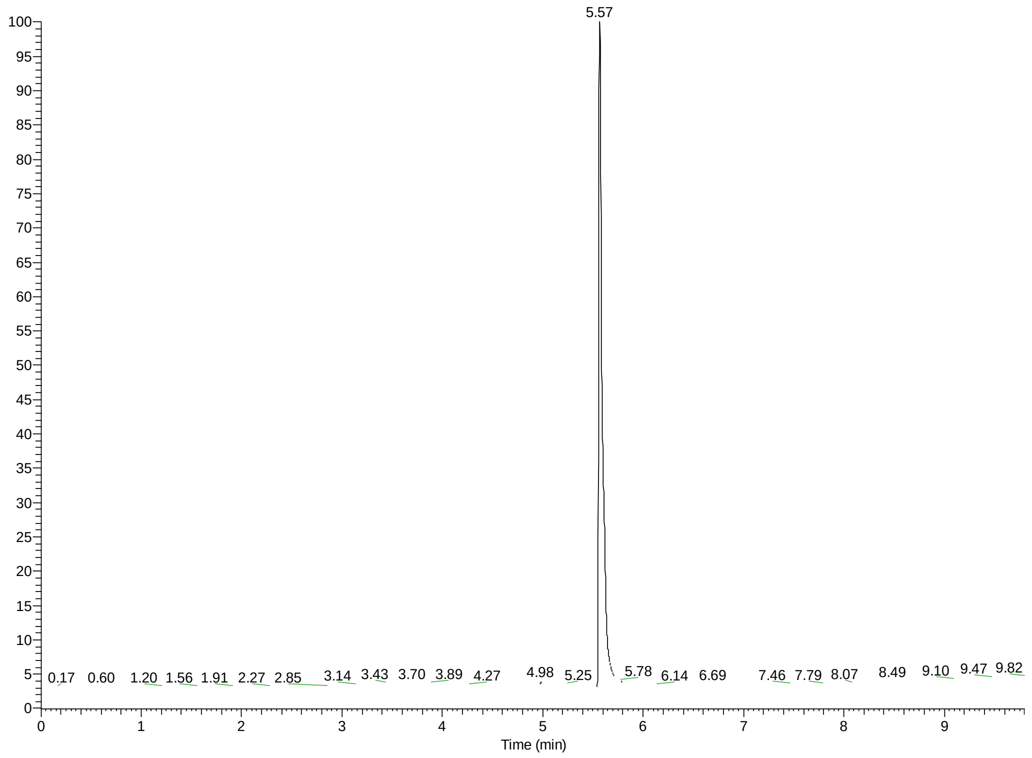

Supplementary Figure S9. Gas chromatography profile of 1-octanol-d_{17}.

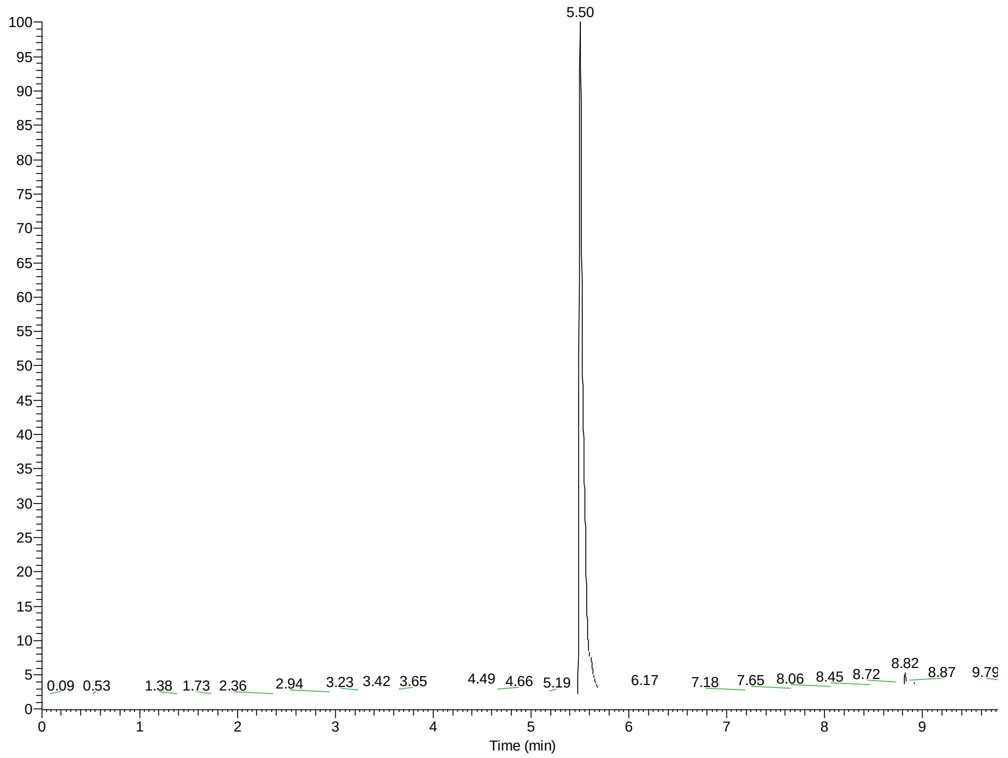

Supplementary Figure S10. Gas chromatography profile of isoamyl acetate.

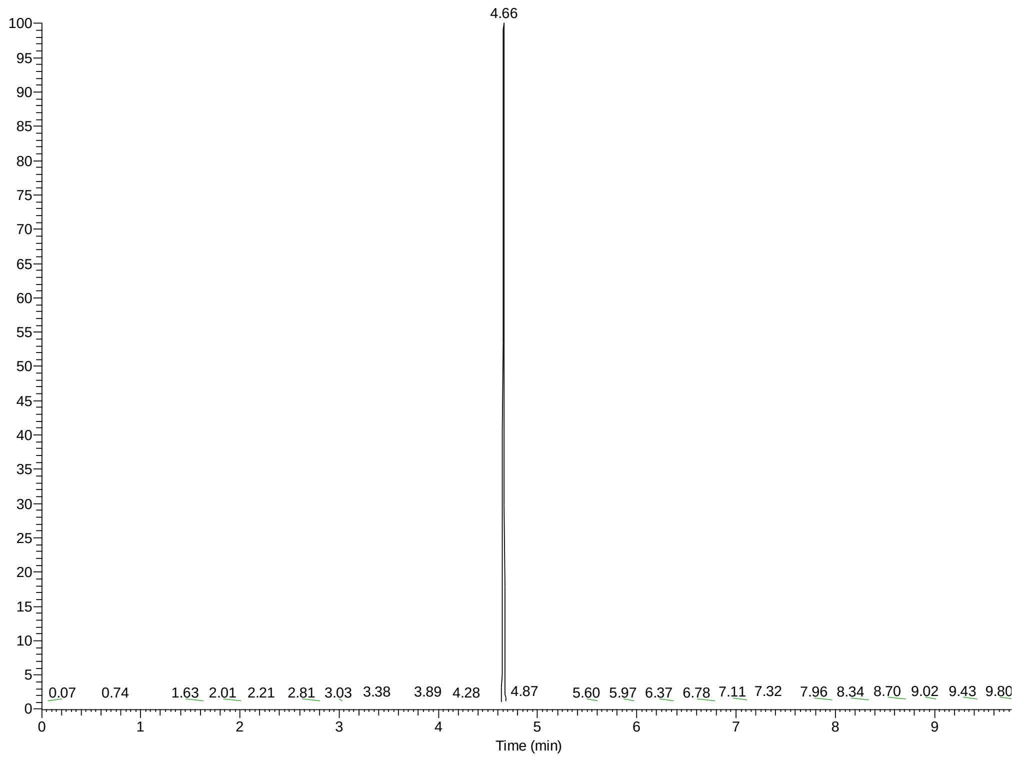

Supplementary Figure S11. Gas chromatography profile of isoamyl acetate-d_3.

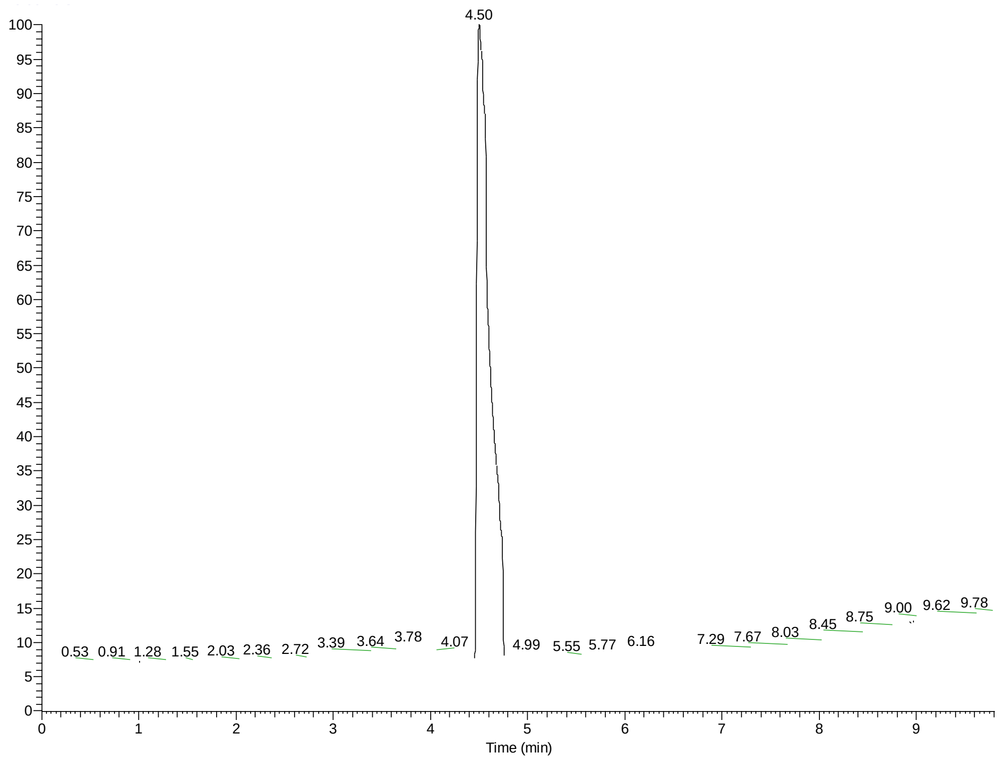

Supplementary Figure S12. 2D plots of principal component analysis. Antennal lobe response dynamics elicited by the odorants during 1s stimulus exposure and 1s post-stimulus and visualized along the first three principal components. Relative variance explained by each principal component is reported in parenthesis. Odorant abbreviations are 1-octanol, OCT; benzaldehyde, BZA; acetophenone, ACP; isoamyl acetate, ISO.

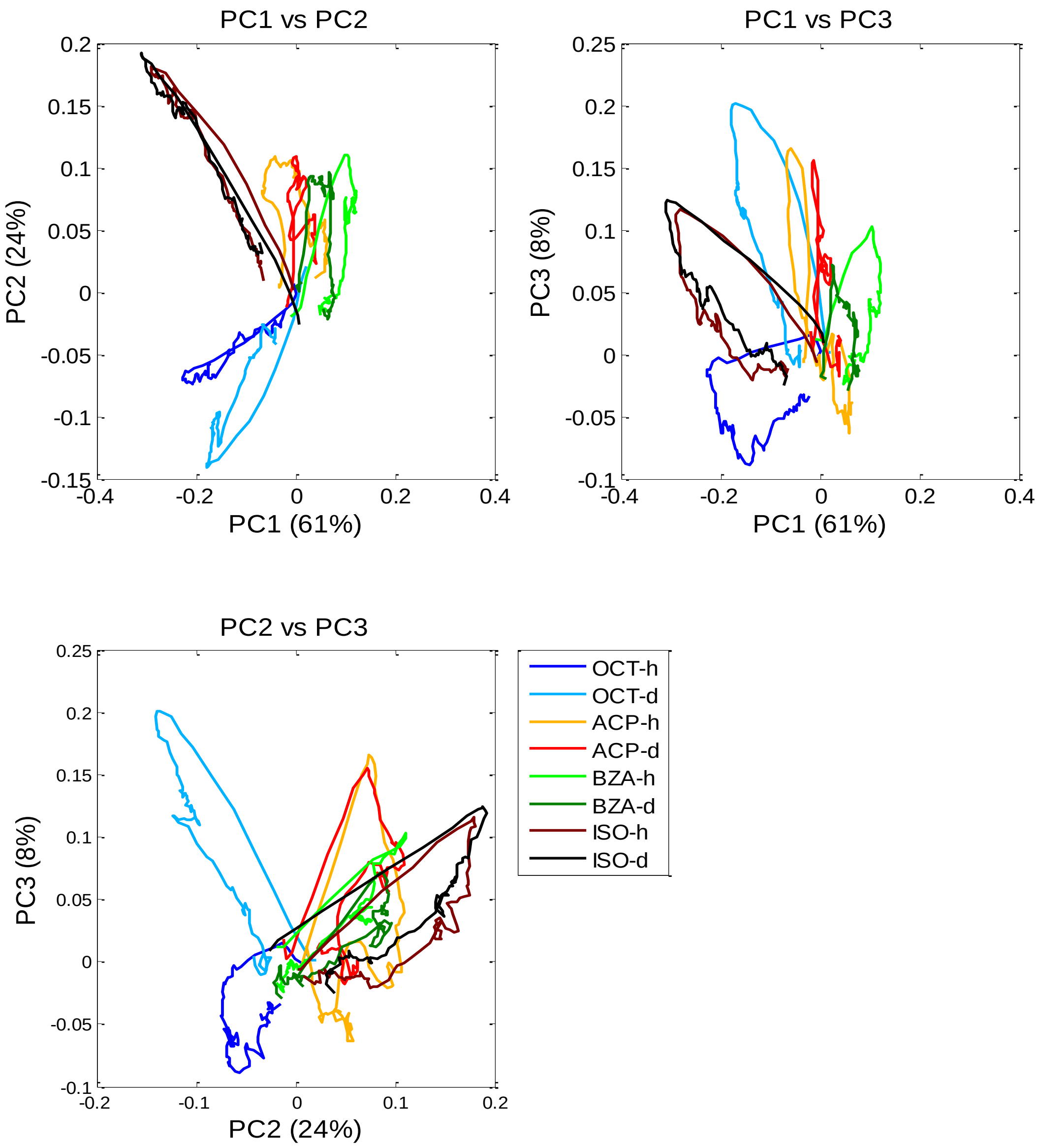

Supplementary Table S1. Gas chromatography analysis of odor vials' headspace.

COMPOUND	% OF PURITY	% OF PRINCIPAL IMPURITY
Acetophenone	99.60	0.16
Acetophenone-d_8	99.89	0.03
Benzaldehyde	99.86	0.07
Benzaldehyde d_5	99.85	0.10
1-octanol	99.28	0.44
1-octanol-d_{17}	99.21	0.79
Isoamyl acetate	99.96	0.02
Isoamyl acetate-d_3	99.29	0.22
